# Effects of teriparatide on cementless bipolar hemiarthroplasty in patients with osteoporotic femoral neck fractures

**DOI:** 10.1186/s12891-016-1149-x

**Published:** 2016-07-19

**Authors:** Tsan-Wen Huang, Kuo-Chin Huang, Shih-Jie Lin, Po-Yao Chuang, Hsin-Nung Shih, Mel S. Lee, Robert Wen-Wei Hsu, Wun-Jer Shen

**Affiliations:** Department of Orthopaedic Surgery, Chang Gung Memorial Hospital, Chiayi, Taiwan No. 6, West Section, Chia-Pu Road, Pu-Tz City, Chia-Yi Hsien 613 Taiwan; Department of Orthopaedic Surgery, Kaohsiung Chang Gung Memorial Hospital, 123 Dapi Road, Niaosong Dist., Kaohsiung City, 83301 Taiwan; Department of Orthopaedic Surgery, Chang Gung Memorial Hospital, Linkou, Taiwan; Chang Gung University, Taoyuan, Taiwan; PO CHENG Orthopedic Institute, 100 Bo-ai 2nd Road Zuoying District, Kaohsiung, Taiwan

**Keywords:** Osteoporosis, Teriparatide, Femoral neck fracture, Hip arthroplasty, Implant migration

## Abstract

**Background:**

For osteoporotic femoral neck fractures, suitable bone-implant stability is critical for pain relief, early return to daily activities and reduction of complications. Teriparatide (parathyroid hormone [PTH1-34]) can improve bone-implant stability in some basic studies. However it’s use in osteoporotic femoral neck fractures treated by cementless hemiarthroplasties for the beneficial effects on bone-implant stability is sparse in the literature. The aim of this study was to determine if post-operative teriparatide administration can reduce femoral stem migration and improve early functional recovery and health-related quality of life (HRQoL).

**Methods:**

Between 2010 and 2014, patients with osteoporotic femoral neck fracture who underwent cementless bipolar hemiarthroplasty were included into this retrospective cohort study. Group A included patients treated with cementless bipolar hemiarthroplasty only; Group B patients had additional teriparatide. Demographic data, complications, radiographic and functional outcomes as well as health-related quality of life (HRQoL) were compared.

**Results:**

There were 52 hips in group A (no teriparatide) and 40 hips in group B (patient who received teriparatide). The subsidence of the femoral stem tended to be significantly decreased in the teriparatide group at 6 and 12 weeks post-operatively (*p* = 0.003 and *p* = 0.008, respectively). The Harris Hip Score (HHS) increased significantly from pre-operation to 6 weeks post-operatively and thereafter up to one year in both groups. However, there were no significant differences in terms of subsequent fracture, mortality, HHS, and HRQoL between two groups during the entire study period.

**Conclusions:**

Teriparatide significantly reduces the subsidence of the cementless femoral stem in elderly patients in the early post-operative period, but this benefit does not reflect better functional outcomes and HRQoL. Further prospective randomized large-scale cohort study is warranted for evidence-based recommendations.

## Background

Femoral neck fracture is one of the most common osteoporotic hip fractures [1]. Displaced femoral neck fracture has been treated traditionally with cemented or cementless hemiarthroplasty, with the goal of accomplishing pain relief and early recovery of daily activities, which are critical for avoiding complications [2]. Cementless hemiarthroplasty has gained substantial popularity in past decades as it helps solve problems pertaining to cemented implants [3]. Achieving immediate stability of the implant is vital for the success of cementless hemiarthroplasty. However, decreased bone regenerative capacity and poor bone stock may affect biologic fixation in elderly patients [4].

Bisphosphonate (BP) has been demonstrated to possess anti-fracture efficacy, preventing peri-prosthetic bone loss [5] and lowering the risk of revision surgery after hip arthroplasty [6]. Recent results with the use of zoledronic acid, a long-acting BP, in reducing migration of the femoral stem have been encouraging [7, 8]; however, the incidence of subsidence of the femoral stem remains high among the elderly [7]. Teriparatide (parathyroid hormone [PTH1-34]), approved by the U.S. Food and Drug Administration (FDA) in 2002, promotes the active building of bone mass via stimulation of the proliferation and differentiation of osteoprogenitor cells [9]. Recent animal studies reveal an acceleration of fracture healing and improvement of bone-implant stability in teriparatide-treated animals [10]. In some human trials, teriparatide appears to enhance fracture healing [11], accelerate lumbar postero-lateral fusion [12], lessen the risk of pedicle screw loosening [13], and increase the purchase of the pedicle screws to the bone [14].

In theory, the positive impact of teriparatide on bone-implant stability is important for an early return to daily activities and reducing morbidity and mortality. This retrospective study was aimed to determine if post-operative teriparatide administration can reduce femoral stem migration and improve early functional recovery and health-related quality of life (HRQoL). Additional correlation analyses were also conducted to determine the role of supplementary teriparatide in reducing complications and mortality in osteoporotic femoral neck fracture.

## Methods

### Clinical and demographic data

This retrospective study was approved by the Ethics Committee and Institutional Review Board of the Chang Gung Memorial Hospital (98-1038B), and all patients provided signed informed consent.

All patients who underwent surgery for osteoporotic femoral neck fracture at our institution since 2010 were routinely entered into the osteoporosis management program. Pharmacologic management, including calcium and vitamin D supplements, anti-resorption drugs and osteo-anabolic drugs, was offered. The merits, disadvantages, and risks of osteoporosis treatment were based on the International Osteoporosis Foundation (IOF) Guidelines for Osteoporosis Treatment [15] and were explained to the patients. The choice of osteoporosis treatments was decided by the patients themselves. Drug use information was included in the computerized database.

**Fig. 1:**
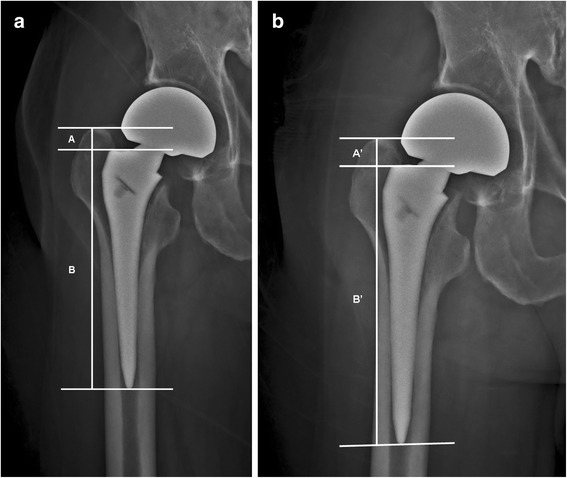
**a** Plain X-ray of the implanted femoral components taken immediately after surgery. The implant (PROFEMUR® Z) fitted at the metaphyseal-diaphyseal junction and the proximal diaphysis. The vertical distance between the lateral shoulder of the prosthesis and the superior tip of the greater trochanter on the radiograph was recorded as A; the distance from the lateral shoulder of the prosthesis to the distal tip of the prosthesis was recorded as B. **b** Plain X-ray of the implanted femoral components taken on follow-up. When measuring subsidence, corrections were made using the ratio of vertical distance between the lateral shoulder of the prosthesis to the distal tip of the prosthesis (B’/B). The corrected vertical distance between the lateral shoulder of the prosthesis and the superior tip of the greater trochanter was calculated using the equation: A X (B’/B). The subsidence was calculated as A’ – A X (B’/B)

We manually reviewed patient records in our database to identify all patients who met the indication of teriparatide treatment (based on IOF Guidelines for Osteoporosis Treatment [15]). To minimize surgeon-related confounding factors, all cementless bipolar hemiarthroplasties were performed by the same surgeon. All patients underwent standardized hemiarthroplasty via a direct lateral approach. On the basis of the standard of care following cementless hemiarthroplasty, the patients were encouraged to ambulate with partial weight-bearing as soon as possible after surgery. To minimize implant-related confounding factors, the patients were treated with the same prosthesis (PROFEMUR® Z hip stem, Wright Medical Technology, Inc. Canadian). The stem had a double-tapered design with grit-blasted surface processing and a rectangular shape that fit at the metaphyseal-diaphyseal junction and proximal diaphysis (Fig. 1a). To minimize drug-related variables, patients who received anti-osteoporotic drugs before surgery were excluded.

Patients with (1) a minimum follow-up of <12 months; (2) multiple fractures, pathologic fractures, previous ipsilateral hip or femur surgery, or fracture of the contralateral hip; (3) musculoskeletal conditions that altered bone conditions, like arthrogryposis multiplex congenita, poliomyelitis, cerebral palsy, developmental abnormality, and Down syndrome; (4) contraindication to the use of teriparatide; (5) complications related to teriparatide use, such as generalized weakness and hypercalcemia; (6) minimum teriparatide treatment course of <12 months; (7) inability to ambulate pre-operatively; and (8) incomplete medical records, radiographic analyses, and clinical functional assessments, were excluded.

The patients were then divided into two subgroups: those who received only calcium and vitamin D supplements (600 mg of calcium and 800 international units (IU) of vitamin D3 per day) (Group A) and those who post-operatively received teriparatide and calcium and vitamin D supplements (Group B). To determine adequate sample size, *a priori* power analysis using the hypothesis test with a power of 80 % and significance of 0.05 was performed. Based on Friedl et al. [7], a pre-study sample size calculation of 24 patients was required in each group to detect a difference in implant migration of 0.5 mm/yr with a power of 80 % at a 5 % significance level. According to Achten et al. [16], 28 hips were required per group to detect a difference of 10 points in the Harris Hip Score (HHS) (estimated Standard deviation of 13). The cut-off value was selected because a difference of 10 points was suggested as the minimal clinically important difference.

### Assessments

#### Radiographic assessment

**Fig. 2:**
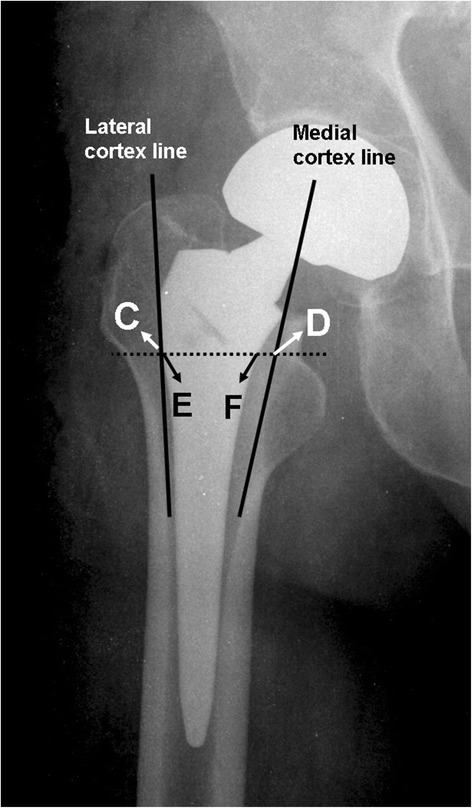
Assessment of the femoral stem-canal ratio. A line was drawn along the lateral margin of the lateral cortex and extended, and then the width between this line and the medial cortex was measured (C-D). Femoral stem-canal ratio was defined as the ratio of the width of the femoral component to the width of the medullary canal (E-F/C-D). Satisfactory was recorded when the ratio was more than 80 %. Radiographic evaluation originally appeared in J Bone Joint Surg Br 1993;75:6-13. E-F: the width of the femoral component

All patients enrolled in this study were assessed by radiographic examination, including antero-posterior (AP) views of the pelvis and lateral views of the affected hip pre-operatively, immediately after surgery and post-operatively at 6 and 12 weeks, 6 and 12 months, and then yearly. Radiographic evaluation of the hip prosthesis was performed with each standard pelvis AP and lateral projection according to the system described by Engh, Massin, & Suthers [17] and Johnston et al. [18]. Using post-operative radiographs, we recorded the position of the femoral stem (valgus, neutral or varus), implant migration (Fig. 1), femoral stem-canal ratio (Fig. 2) [19], radiolucent lines around the prosthesis, and pedestal formation of the unstable stem. A stem migration of 0.6 mm was considered a clinically important difference and was defined as femoral stem subsidence, based on Thomas Baad-Hansen et al. [20].

Bone mineral density (BMD) of the opposite hip was measured using the Hologic DXA QDR 4500 (Hologic Inc., Waltham, MA) after surgery. The radiographic assessments and BMD of the opposite hip were reviewed and analyzed by an independent surgeon who was blinded to the groupings and patient demographic data. Intra-observer reliability was assessed according to the method described by Konigsberg et al. [21], and rated as good to very good.

#### Functional assessment

Pre-operative surgical risk was categorized according to the classification of the American Society of Anesthesiologists (ASA). Functional assessments including the HHS and Short-Form-12 (SF-12) were performed at pre-injury and at post-operative 3, 6, 9, and 12 months. The SF-12 is a HRQoL scale composed of 12 questions. Physical Component Summary (PCS) and Mental Component Summary (MCS) scores were estimated by the assignment of different weights to the questions. A higher score was related to a better outcome. All functional outcomes were assessed by an independent observer who was also blinded to the groupings and patient demographic data.

Complications and use of analgesics were recorded. Any medical or surgical event that compromised the clinical recovery of patients, such as wound infection, pneumonia, urinary tract infection, venous thrombo-embolism (VTE), neurovascular injury, fracture, dislocation, implant malposition, implant size mismatch, or early loosening, was defined as an adverse event. A relatively poorer and slower functional recovery beyond 3 months and an HHS score <80 were also categorized as adverse events.

### Statistical analyses

All data were collected and independently entered into a Microsoft Excel spreadsheet by an independent observer. After the spreadsheets were re-checked for missing and illogical data, the data were copied into SPSS version 13.0 for Windows (SPSS Inc., Chicago, IL) and analyzed. Student’s t test was used to compare the variables of age, body mass index, hospital stay, BMD of contralateral hip, femoral stem-canal ratio, follow-up time, subsidence of stem, and functional results. Between-group comparisons of continuous variables at each time point were analyzed using the Mann-Whitney U test, while within-group comparisons between time points and baseline values were performed using the Wilcoxon signed-rank test. Categorical data were analyzed using the chi-square test and Fisher’s exact test, as appropriate. All data were analyzed by an independent statistician who was blinded to the grouping, patient demographic data, and function outcomes. Significance was set at *p* < 0.05.

## Results

Between 2010 and 2014, 112 patients (112 hips) met our inclusion criteria. However, 14 patients who used anti-osteoporotic drugs prior to surgery and 6 with incomplete data were excluded, leaving 92 patients (92 hips), including 52 in Group A (no teriparatide) and 40 in Group B (treated with teriparatide), for further analysis. The mean follow-up time was 38.4 months (range, 12–59 months). There were no differences in age, body mass index, ASA classification, hospital stay, BMD of the contralateral hip, femoral stem-canal ratio, and follow-up time between the two groups (Table 1).

**Table 1:** Demographic Data of the Teriparatide and Control Groups

Parameters	Group A	Group B	*p* value
	*N* = 52	*N* = 40	
*Variables*			
Age at time of operation (yrs)	71.0 ± 8.3	72.1 ± 7.6	0.682
Body mass index (kg/m2)	26.9 ± 2.5	25.9 ± 3.1	0.693
ASA classification			0.829
ASA I	-	-	-
ASA II	33 (63.5 %)	24 (60.0 %)	
ASA III	19 (36.5 %)	16 (40.0 %)	
Hospital stay (day)	7.3 ± 1.6	6.9 ± 1.6	0.464
BMD of contralateral hip (T-score)	−4.3 ± 1.2	−4.2 ± 1.0	0.908
Femoral stem-canal ratio (%)	88.3 ± 5.2	87.6 ± 5.3	0.620
Follow-up (months)	27.1 ± 7.3	26.2 ± 7.4	0.650

Group A: patients without supplementary pharmacologic treatment

Group B: patients treated with teriparatide

Values are shown as mean (standard deviation) or as the n (%)

*p*-values for between-group comparisons were determined by the chi-squared test and Fisher’s exact test for categorical variables and Student’s t test for continuous variables

*Statistically significant (*p* < 0.05)

There was no difference between complications of the two groups, except for subsidence rates (*p* = 0.028). A summary of complications during the 12-month follow-up revealed no deep wound infection, VTE, intra-operative peri-prosthetic fracture, early loosening of the femoral stem, or dislocation had occurred in either group (Table 2). Both groups showed similar findings with regard to superficial wound infection, pneumonia, urinary tract infection, and post-operative peri-prosthetic fracture. Six patients in Group A and four patients in Group B died during the follow-up period due to reasons unrelated to the operation (*p* = 0.545). The overall complication rate between the two groups was significantly different (*p* = 0.020) (Table 2).

**Table 2:** Post-operative Complications in the Teriparatide and Control Groups

Parameters	Group A	Group B	*p* value
	*N* = 52	*N* = 40	
*Variables*			
Superficial wound infection	2 (3.8 %)	1 (2.5 %)	0.598
Deep wound infection	0	0	-
Pneumonia	2 (3.8 %)	1 (2.5 %)	0.598
Urinary tract infection	2 (3.8 %)	1 (2.5 %)	0.598
Venous thrombo-embolism	0	0	-
Mortality	6 (11.5 %)	4 (10 %)	0.545
Intra-operative peri-prosthetic fracture	0	0	-
Post-operative peri-prosthetic fracture	0	1 (2.5 %)	0.435
Subsidence of the femoral stem	18 (34.6 %)	6 (15.0 %)	0.028*
Early loosening	0	0	-
Dislocation	0	0	-
Overall complications	30 (57.7 %)	14 (35.0 %)	0.020*

Group A: patients without supplementary pharmacologic treatment

Group B: patients treated with teriparatide

The values are given as the n (%)

*p*-values for between-group comparisons were determined by the chi-squared test and Fisher’s exact test

*Statistically significant (*p* < 0.05)

Twenty-three patients (13 in Group A and 10 in Group B) were excluded from the final analysis: ten patients died (6 in Group A and 4 in Group B), one had post-operative peri-prosthetic fracture (Group B), and 12 had sustained subsequent fracture, including vertebral fracture in 6 patients (4 in Group A and 2 in Group B), hip fracture in 2 (one in Group A and one in Group B), and wrist fracture in 4 (2 in Group A and 2 in Group B) (Table 3). Patients who sustained peri-prosthetic fracture and subsequent fracture were further excluded because there would be different rehabilitation protocols and influences on outcome assessment. The remaining 69 patients (39 in Group A and 30 in Group B) were included in the final analysis.

**Table 3:** Subsequent Fracture in the Teriparatide and Control Groups during the 12-month Follow-up

Parameters	Group A	Group B	*p* value
	*N* = 52	*N* = 40	
*Subsequent fracture*			
Vertebral fracture	4 (7.7 %)	2 (5.0 %)	0.470
Hip fracture	1 (1.9 %)	1 (2.5 %)	0.683
Wrist fracture	2 (3.8 %)	2 (5.0 %)	0.587
Overall subsequent fractures	7 (13.5 %)	5 (12.5 %)	0.574

Group A: patients without supplementary pharmacologic treatment

Group B: patients treated with teriparatide

The values are given as the n (%)

*p*-values for between-group comparisons were determined by the chi-squared test and Fisher’s exact test

*Statistically significant (*p* < 0.05)

**Fig. 3:**
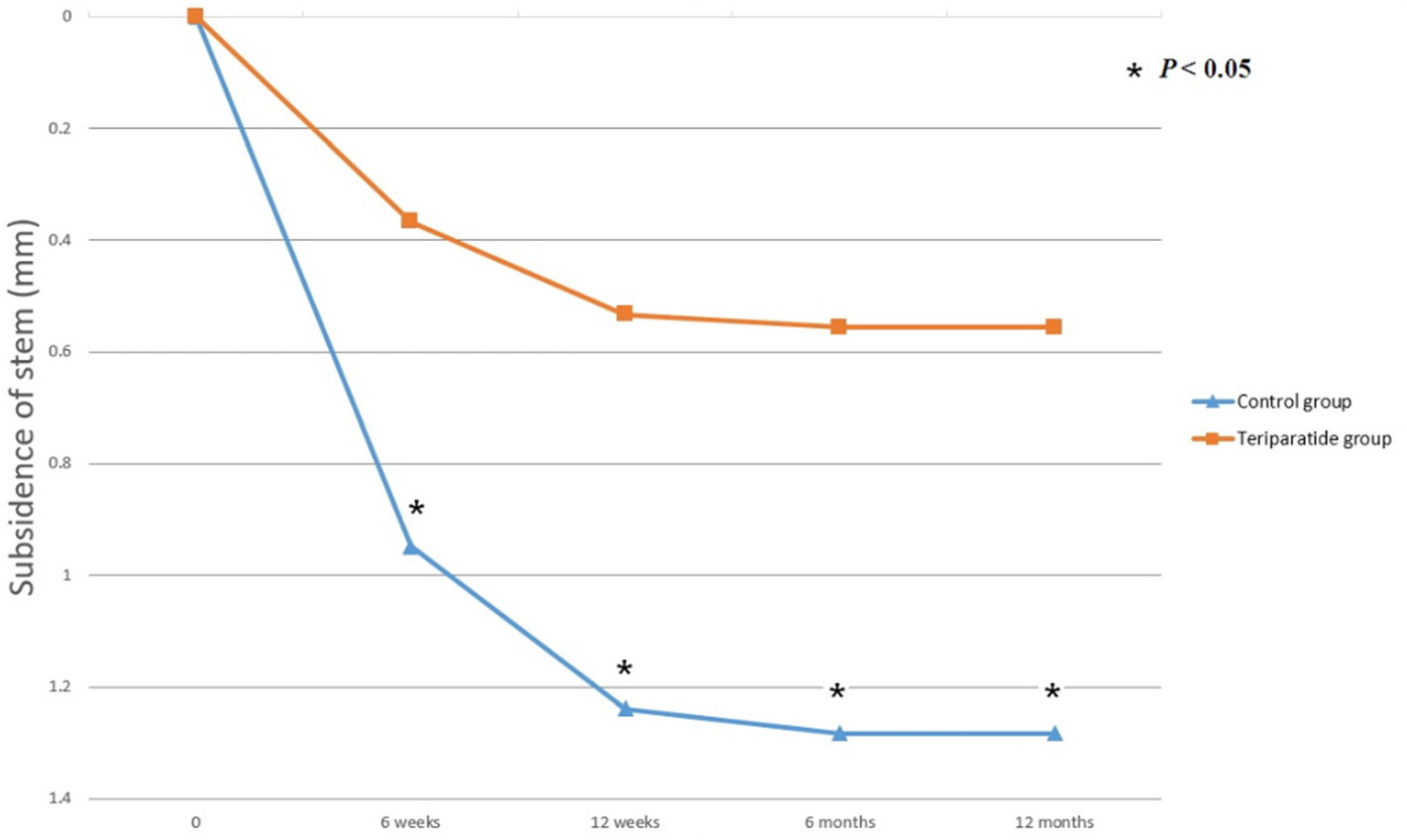
The migration pattern of the femoral components. The values are the mean for the teriparatide and control groups on follow-up intervals immediately after surgery and at 6 and 12 weeks and 6 and 12 months. The subsidence of the stem was significantly decreased in the teriparatide group compared to the control group at each time point. The distal migration of the stem reached a plateau phase at 6 months and 12 months post-operatively in both groups. *p < 0.05, Mann-Whitney U test

In the radiographic analysis, subsidence of the stem in Group A significantly increased up to −1.0 ± 0.9 mm and −1.3 ± 1.2 mm at 6 and 12 weeks, respectively (Fig. 3). However, the migration pattern reached a plateau at 6 and 12 months. In the final analysis, all implants showed stable osteo-integration without evidence of further migration. There were no radiolucent lines at the prosthesis-bone interface and no pedestal formation in any stem in either group. The HHS increased significantly from pre-operation to 6 weeks post-operatively and thereafter up to one year in both groups. The differences in scores, however, did not achieve statistical significance between the two groups throughout the study period (Table 4). The two groups did not differ in SF-12 (PCS) and SF-12 (MCS) during the entire study period (Table 5).

**Table 4:** Functional Outcomes Assessments at Different Time Intervals

	Pre-injury	Post-operation			
		3 months	6 months	9 months	12 months
*HHS score*					
Group A	15.9 ± 9.1	56.2 ± 14.1	70.9 ± 13.0	77.9 ± 10.6	83.6 ± 7.5
Group B	15.4 ± 8.4	58.8 ± 10.1	71.1 ± 12.3	79.9 ± 9.4	81.5 ± 9.9

Group A: patients without supplementary pharmacologic treatment

Group B: patients treated with teriparatide

Values are shown as mean (standard deviation)

*p*-values for between-group comparison were determined by Student’s t test

Abbreviations: HHS, Harris hip score

*Statistically significant (*p* < 0.05)

**Table 5:** Health-Related Quality of Life (HRQoL) Assessments at Different Time Intervals

	Pre-injury	Post-operation			
		3 months	6 months	9 months	12 months
*SF-12 PCS*					
Group A	45.9 ± 8.5	28.2 ± 11.3	37.2 ± 10.8	39.2 ± 11.0	44.3 ± 11.6
Group B	46.2 ± 9.8	29.2 ± 11.3	38.1 ± 10.6	40.3 ± 9.9	45.0 ± 10.0
*SF-12 MCS*					
Group A	59.2 ± 11.9	50.8 ± 11.1	53.7 ± 9.3	51.9 ± 10.6	54.2 ± 10.5
Group B	58.6 ± 10.9	51.7 ± 11.6	53.4 ± 9.5	52.4 ± 11.9	54.5 ± 10.9

Group A: patients without supplementary pharmacologic treatment

Group B: patients treated with teriparatide

Values are shown as mean (standard deviation)

*p*-values for between-group comparison were determined by Student’s t test

Abbreviations: HHS, Harris hip score

*Statistically significant (*p* < 0.05)

## Discussion

The most important finding in this investigation is that teriparatide use lessens the subsidence of cementless femoral stems compared to non-use of teriparatide in the early post-operative period (6 and 12 weeks, post-operatively). However, this benefit does not translate to better functional outcomes and HRQoL. There was no statistically significant difference between the two groups in subsequent fracture and mortality.

Osteoporotic femoral neck fractures often contribute to pain and immobility and lead to a loss of ability to perform daily activities and a reduction in quality of life, which are associated with high morbidity and mortality [22, 23]. The annual number of these fractures worldwide, as estimated by the IOF, will be as high as 4.6 million by 2025 and 6.26 million by 2050 [23]. This injury is relatively common and is a serious clinical issue affecting the elderly; it is a burden on individuals and on the health-care system, as well as society in general [1, 22, 23]. The primary treatment goals are pain relief, improvement in mobilization and the prevention of complications, which are of critical importance [1, 2]. However, this is also a challenge for orthopedic surgeons because of decreased bone regenerative capacity and poor bone stock [4].

In treating osteoporotic femoral neck fractures, the orthopedic surgeon is often the first physician to address the injury and is in a unique position to make every effort to treat, prevent subsequent fractures, and minimize the need for subsequent revision surgery [1, 2, 5–8]. Since bipolar hemiarthroplasty is commonly performed in elderly patients with femoral neck fracture, post-operative administration of anti-osteoporotic drugs is reasonable and may play a valuable role in the treatment [1, 2]. Among these anti-osteoporotic drugs, BP is widely used and has been demonstrated to prevent peri-prosthetic bone loss [5, 8, 24], lower the risk of revision surgery after hip arthroplasty [6], and reduce femoral stem migration [7, 8]. However, subsidence of the femoral stem is still higher in elderly patients [7]. Moreover, concerns exist regarding BP-induced osteonecrosis of the jaw, atypical femoral fracture, and atypical peri-prosthetic fracture after long-term BP treatment [25, 26].

Osteoblast activity may play an important role in osteo-integration between bone and prosthesis [27]. The anabolic agent teriparatide was approved by the U.S. FDA in 2002, and was indicated for the treatment of osteoporosis in men and in post-menopausal women [1]. Unlike anti-resorption drugs, teriparatide was found to directly stimulate osteoblasts to increase trabecular connectivity, cortical thickness, and bone mineral content in the peri-implant area, thereby accelerating fracture healing and lumbar postero-lateral fusion and improving bone-implant stability in treated animals [9, 10] and in human trials [11–14]. In animal studies, finite element analysis, and human trials, teriparatide showed superiority over other BPs in improving femoral strength, mechanical fixation of implants, and bone volume around the implant [28, 29]. Despite the higher cost and relatively poor compliance compared to other anti-osteoporotic drugs, teriparatide may be a reasonable supplemental treatment to enhance the stability of cementless femoral stems in elderly patients after hemiarthroplasty for femoral neck fracture. However, there is no published report on the role of teriparatide in this situation.

The beneficial effects of teriparatide on implant fixation have been reported recently [13, 14]. Ohtori S et al. [13] prospectively studied 62 post-menopausal women and reported that teriparatide increased the quality of lumbar spine bone marrow and pedicle cortex. There was also a significantly lower incidence of pedicle screw loosening in the teriparatide group than in the risedronate or control groups. Inoue et al. [14] reported a cohort study of 29 post-menopausal women who underwent fusion surgery of the thoracic and/or lumbar spine and concluded that pre-operative teriparatide treatment might be an option for maximizing adherence of the pedicle screws to the bone at the time of fusion surgery. This study retrospectively analyzed the effect of teriparatide in preventing subsidence of the cementless femoral stem in elderly patients after bipolar hemiarthroplasty for femoral neck fracture. Teriparatide significantly reduced femoral stem migration compared to a control group of patients with calcium and vitamin D supplements only.

Since peri-prosthetic bone loss is most pronounced in the early post-operative period [30], decreases in bone regenerative capacity in the elderly may impact osteo-integration at the prosthesis-bone interface and further contribute to subsidence of the femoral stem. The absence of osteo-integration of the femoral stem is related to micro-movements at the prosthesis-bone interface. In this study, the adequacy of fit and fill of the femoral components in the medullary canal was examined, and all femoral stems filled the canal by more than 80 %. Moreover, patients were allowed protection against weight-bearing due to a concern about subsidence that could result from inadequate osteo-integration. However, significant subsidence of the femoral stem still occurred in the control group.

Improvements in osteo-integration at the prosthesis-bone interface could allow elderly patients with femoral neck fracture to return to their daily activities and reduce morbidity and mortality. However, this study found no statistically significant differences in clinical outcomes, HRQoL, subsequent fracture, and mortality between those with and those without supplemental teriparatide treatment. The most likely reason for this is that migration of the prosthesis was limited in the control group. The femoral stem subsided initially, but stabilized at post-operative 12 weeks (Fig. 3).

This study has several limitations. First, it was a retrospective study, with all the inherent weaknesses and biases of such a study design. Teriparatide is approved by the Taiwanese FDA for treatment of osteoporosis only. Although evidence from studies utilizing animal models indicates teriparatide can improve osteo-integration at the prosthesis-bone interface, this use in humans is “off label” and it is difficult to obtain approval for a prospective study from the Ethics Committee and Institutional Review Board. Second, the patient number was small. Patients who took anti-osteoporotic drugs prior to surgery were excluded to avoid the confounding factors of medication. A single surgeon performed all hemiarthroplasties and the same post-operative rehabilitation protocol was used. The same prosthesis was implanted to avoid implant-related confounding factors. The strict inclusion criteria were meant to limit the study variables, but this also reduced the numbers of subjects and limited the power of the study to detect clinically significant differences. Although adequate sample sizes were determined to detect differences in subsidence of the femoral stem [7] and HHS [16], this study still may be underpowered to demonstrate significant differences. Third, in treating osteoporotic femoral neck fractures, the orthopedic community is divided between those using cementless hemiarthroplasty and those using cemented hemiarthroplasty. In order to avoid implant-related confounding factors, the current study focused on treatment with cementless hemiarthroplasty. Thus, the findings cannot be applied to teriparatide treatment with cemented hemiarthroplasty. Fourth, there are two different recombinant PTHs (PTH1-34 and PTH1-84), but only teriparatide (recombinant PTH1-34) is available in Taiwan. Although recombinant PTH1-84 also has had a clinical effect on fracture healing in post-menopausal women [31], similar results with its use cannot be assumed. There are also no comparative studies of the anabolic effects of the two drugs. Last, although differences between the two groups got statistical significance, differences between mean value of subsidence in the two groups is only about 0.7 mm. Clinical importance of this difference is limited. Meanwhile, measurements of the X-ray can not be so accurate, and this will also reduce the importance of the difference.

## Conclusions

This study found a significant reduction of subsidence of the cementless femoral stem in elderly patients with femoral neck fractures who were provided teriparatide treatment. But teriparatide provided no significant benefits in terms of clinical outcome, HRQoL, subsequent fracture, and mortality. Further prospective randomized large-scale cohort studies are warranted to determine if the reduction in femoral stem subsidence will translate into significantly superior clinical outcomes and HRQoL, as a basis for evidence-based recommendations.

## Abbreviations

AP, antero-posterior; ASA, American Society of Anesthesiologists; BMD, bone mineral density; BP, bisphosphonate; FDA, Food and Drug Administration; HHS, Harris Hip Score; HRQoL, health-related quality of life; IOF, International Osteoporosis Foundation; MCS, Mental Component Summary; PCS, Physical Component Summary; PTH, parathyroid hormone; SF-12, Short-Form-12; VTE, venous thrombo-embolism
